# Supplementary material for: Modulation between capacitor and conductor for a redox-active 2D bis(terpyridine)cobalt(II) nanosheet via anion-exchange
Source: Commun Chem. 2024 Aug 22;7:186. doi: 10.1038/s42004-024-01274-4 (PMC11341730; doi:10.1038/s42004-024-01274-4)
Supplement: Supplementary file 2 — Supplementary Material [file 42004_2024_1274_MOESM2_ESM.pdf]

Supplementary Material for

Modulation between Capacitor and Conductor for a Redox-Active 2D Bis(terpyridine)cobalt(II) Nanosheet via Anion-Exchange

Kenji Takada,[1]* Miyu Ito,[2] Naoya Fukui,[1] Hiroshi Nishihara[1,2]*

[1]*Research Institute for Science and Technology, Tokyo University of Science, 2641, Yamazaki, Noda, Chiba 278-8510, Japan*

[2]*Faculty of Science and Technology, Tokyo University of Science, 2641, Yamazaki, Noda, Chiba 278-8510, Japan*

E-mail addresses: takada.k.ag@rs.tus.ac.jp, nisihara@rs.tus.ac.jp

Index

A. Characterization of **1**
B. Electrochemistry of **1**
C. Chemical structures of anions
D. Cross-sectional SEM/EDS analysis of **2** and **3**
E. XP spectra of **1−3**
F. UV-vis spectra of (nBu$_4$N)$_n$[Ni(mnt)$_2$] (n = 1, 2)
G. Thickness of **1−3**
H. Raman spectra of (nBu$_4$N)$_n$[Ni(mnt)$_2$] (n = 1, 2)
I. Inverse anion-exchange reaction of **2** and **3**
J. Optical microscopy image of **1** on IDA
K. Electrochemical impedance spectroscopy for **1−3**
L. SEM/EDS analysis of **4**
M. XPS of **4**
N. Raman spectra of **4**
O. Electronic conductivity measurement of **4**
P. Conductivity measurement of **2**
Q. Potential-dependent conductivity of **2**
R. Electrochemistry of **2** and **3**
S. Supplementary references

A. Characterization of **1**

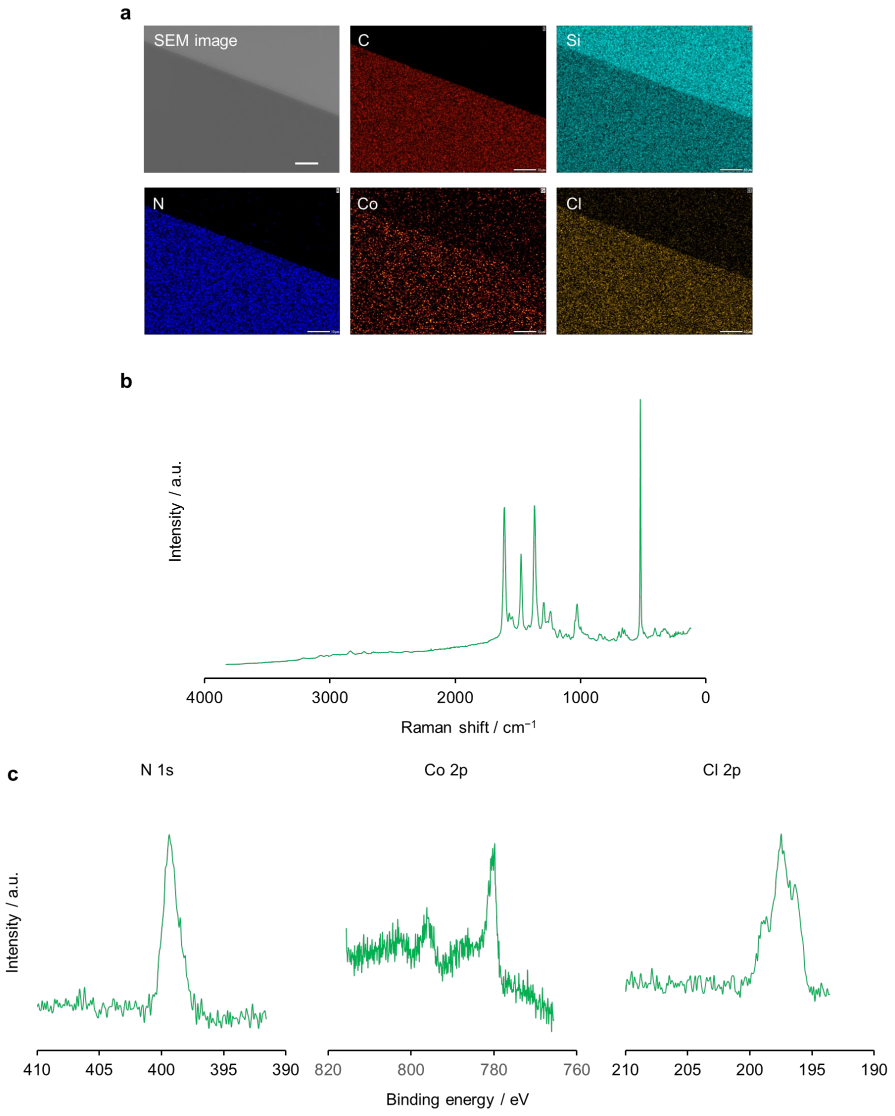

Supplementary Figure 1. Characterization of **1**. (**a**) SEM/EDS of **1**. (**b**) Raman spectrum of **1**. (**c**) XP spectra of **1** in N 1s, Co 2p and Cl 2p core levels.

B. Electrochemistry of **1**

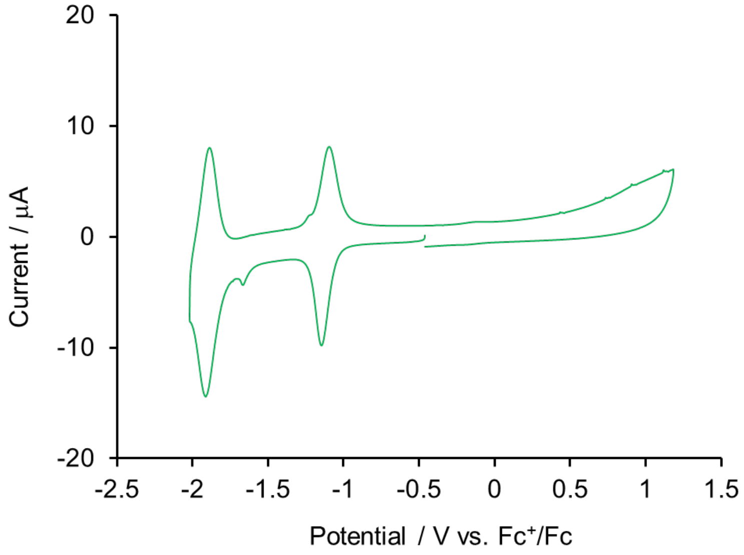

Supplementary Figure 2. Cyclic voltammogram of **1**. (1 M *n*Bu_4NPF_6 in CH_3CN; Scan rate: 50 mV s^{-1})

C. Chemical structures of anions

Supplementary Table 1. Chemical structures of anions encapsulated in **1**−**4**.

Compound	Anion
**1**	Cl^{-}
**2**	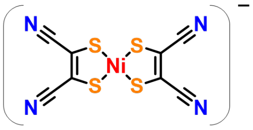
**3**	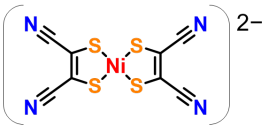
**4**	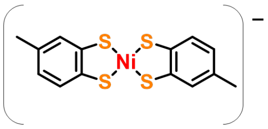

D. Cross-sectional SEM/EDS analysis of **2** and **3**

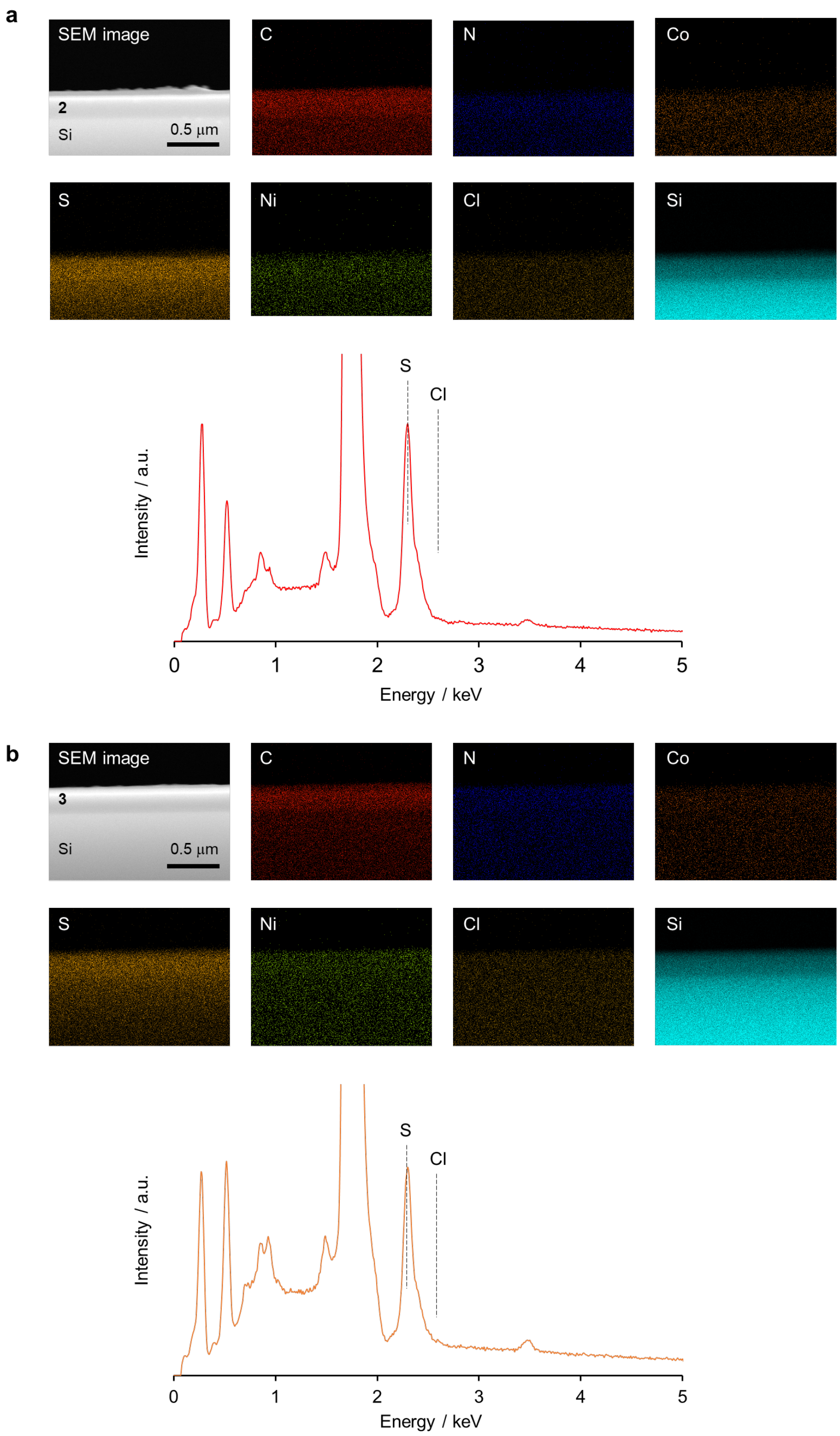

Supplementary Figure 3. Cross-sectional SEM/EDS elemental mapping and spectra of **2** (**a**) and **3** (**b**).

E. XP spectra of **1−3**

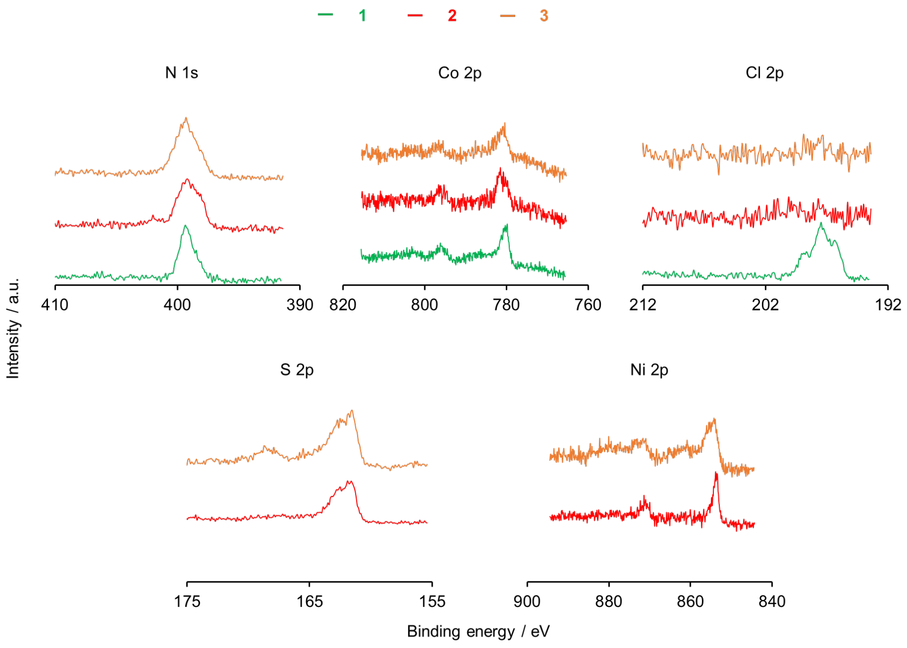

Supplementary Figure 4. XP spectra of **1−3** with N 1s, Co 2p, Cl 2p, S 2p, and Ni 2p core levels.

F. UV-vis spectra of (*n*$Bu_4N)_n[Ni(mnt)_2]$ (n = 1, 2)

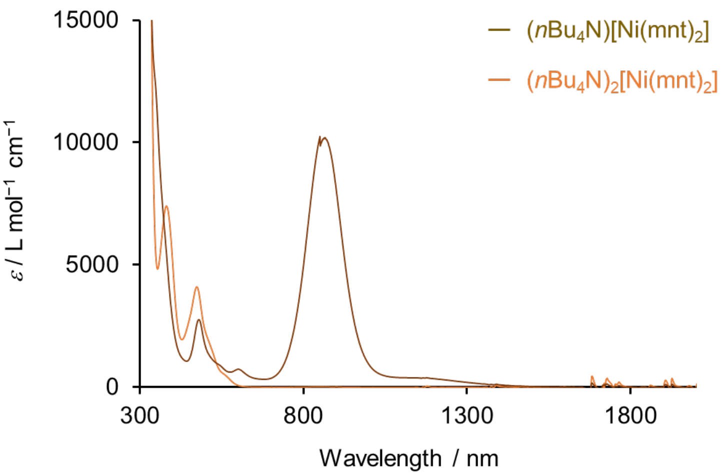

Supplementary Figure 5. UV-vis absorption spectra of (*n*$Bu_4N)[Ni(mnt)_2]$ and (*n*$Bu_4N)_2[Ni(mnt)_2]$ in CH_3CN.

G. Thickness of **1−3**

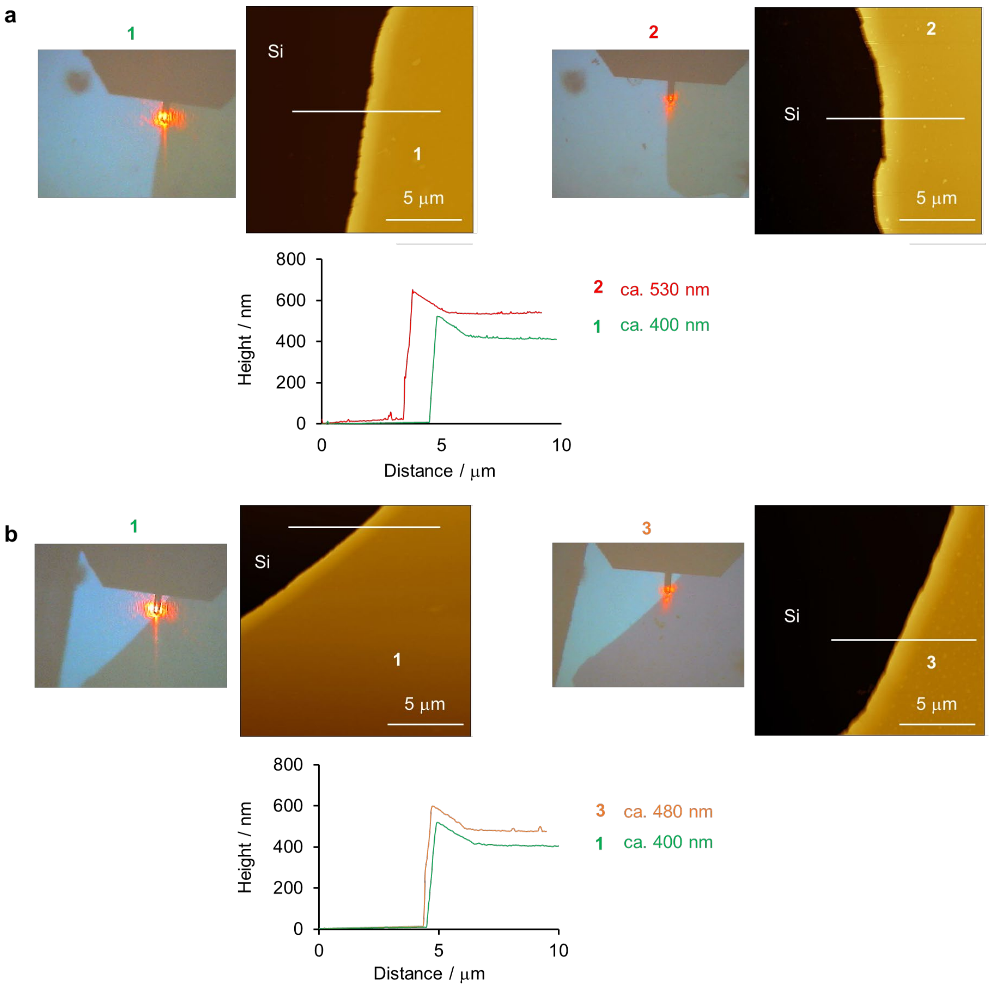

Supplementary Figure 6. AFM images of **2** (**a**) and **3** (**b**). The pictures on the left of AFM images depict the measured area. For the comparison, AFM images both before and after the anion-exchange reaction were recorded at the neighbouring areas. The Height profiles were recorded along the white lines in the corresponding AFM images.

H. Raman spectra of (nBu$_4$N)$_n$[Ni(mnt)$_2$]

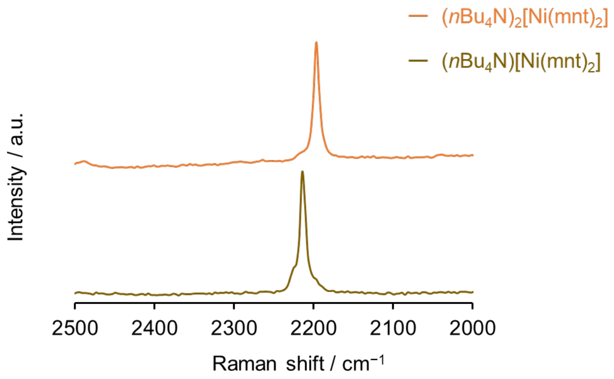

Supplementary Figure 7. Raman spectra of [Ni(mnt)$_2$]$^{n-}$ focusing on the C≡N stretching vibration mode. The peak position of the C≡N stretching vibration is the indicator for the oxidation state of [Ni(mnt)$_2$]$^{n-}$ anions.

I. Inverse anion-exchange reaction of **2** and **3**

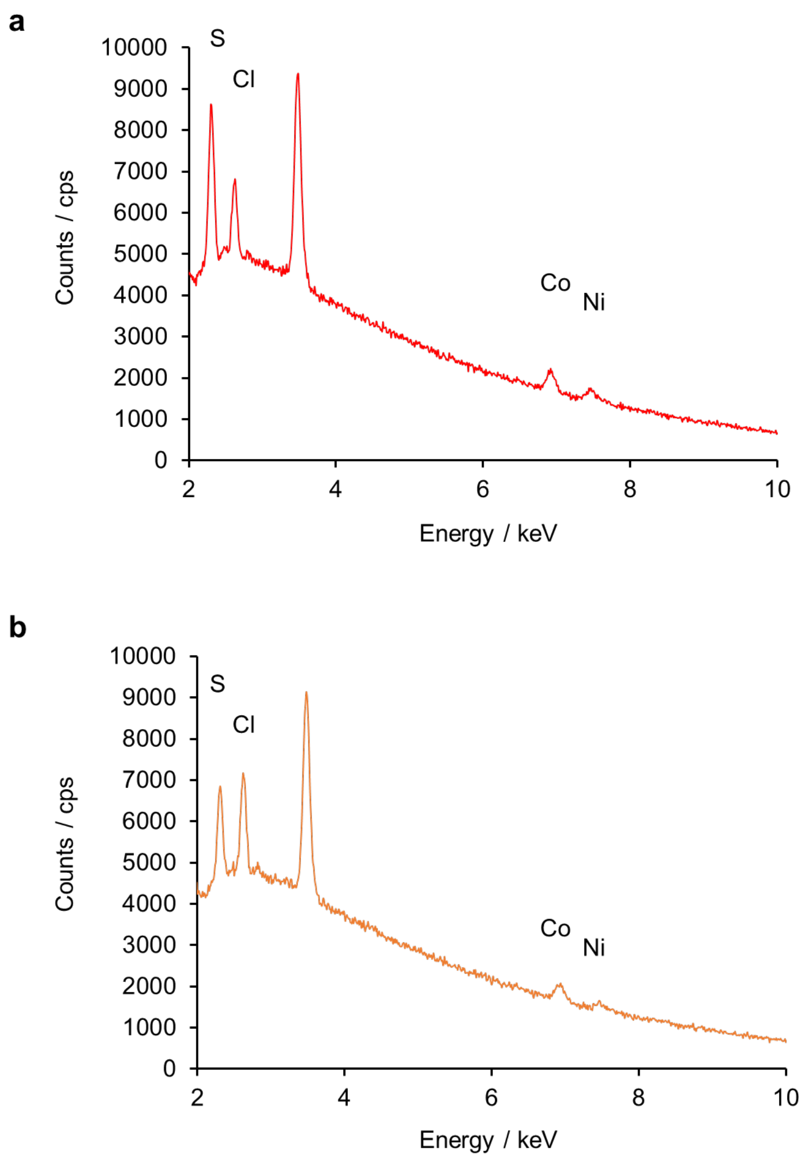

Supplementary Figure 8. SEM/EDS spectra after the inverse anion-exchange with Cl^- for **2** (**a**) and **3** (**b**).

J. Optical microscopy image of **1** on IDA

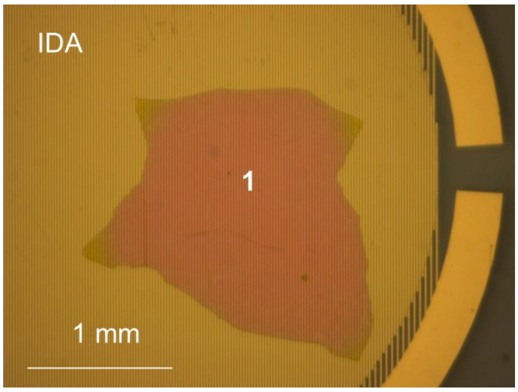

Supplementary Figure 9. Optical microscope image of **1** on Au IDA.

K. Electrochemical impedance spectroscopy for **1**

a

b

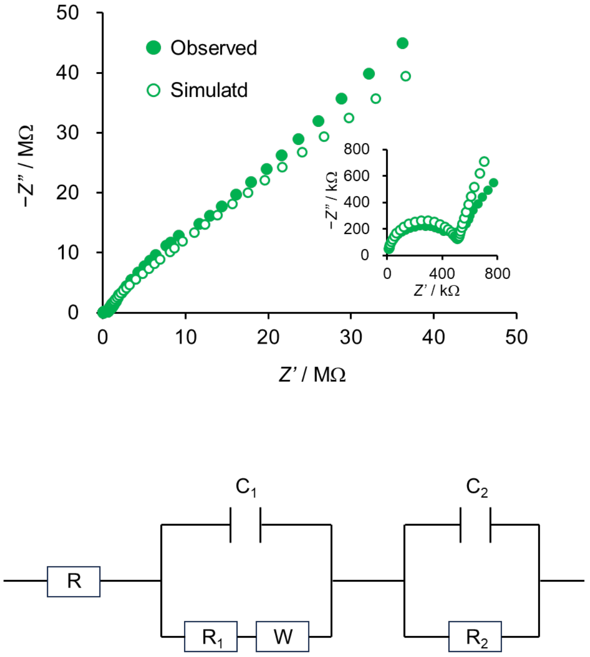

Supplementary Figure 10. Electrochemical impedance spectroscopy of **1** on Au IDA. (**a**) Nyquist plots for experimental (filled circle) and simulated (blank circle) spectra (inset: EIS spectra for high frequency region.). The EIS spectrum was obtained at 0 V with the AC amplitude of 0.05 V. (**b**) Equivalent circuit used for the simulation in **a**. The parameters for each component are followings: R = 8687 Ω, R_1 = 294 Ω, $C_1 = 1.84\times10^{-9}$ F, W = 2.26×10^{-8} Ω, $R_2 = 5.04\times10^{5}$ Ω, $C_2 = 1.84\times10^{-11}$ F.

L. SEM/EDS analysis of **4**

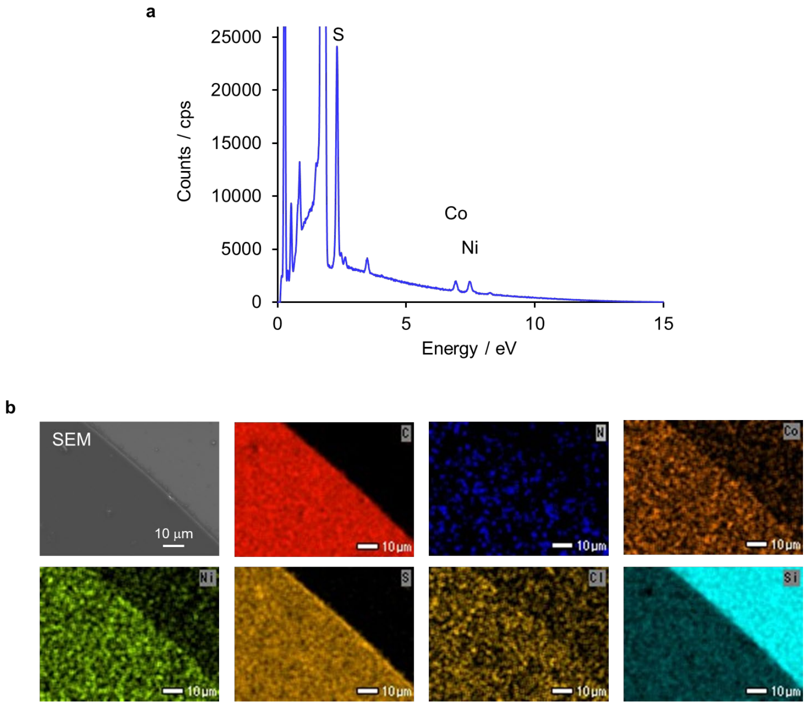

Supplementary Figure 11. SEM/EDS analysis of **4**. (**a**) EDS spectrum and (**b**) elemental mapping.

M. XPS of **4**

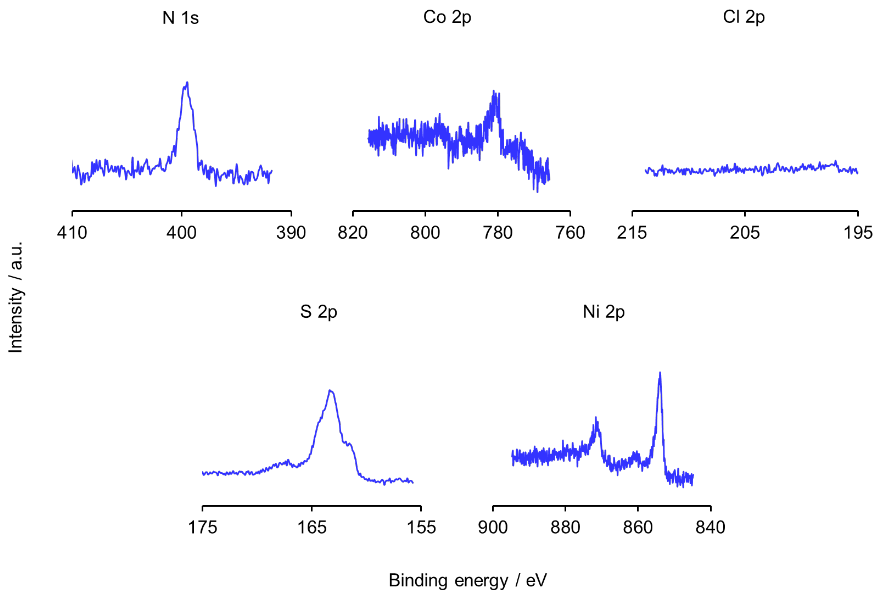

Supplementary Figure 12. XP spectra of **4** in N 1s, Co 2p, Cl 2p, S 2p, and Ni 2p core levels.

N. Raman spectrum of **4**

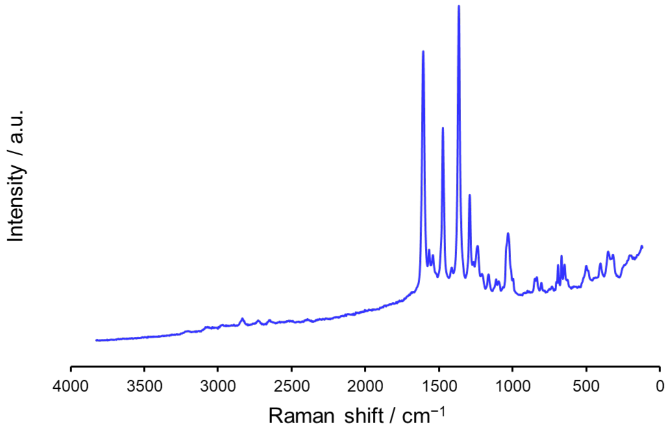

Supplementary Figure 13. Raman spectra of **4**.

O. Electronic conductivity measurement of **4**

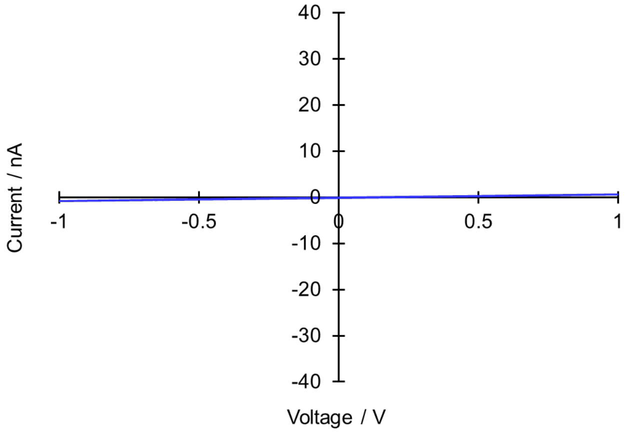

Supplementary Figure 14. An *I*-*V* curve for **4** between −1 V and +1 V.

P. Conductivity measurement of **2**

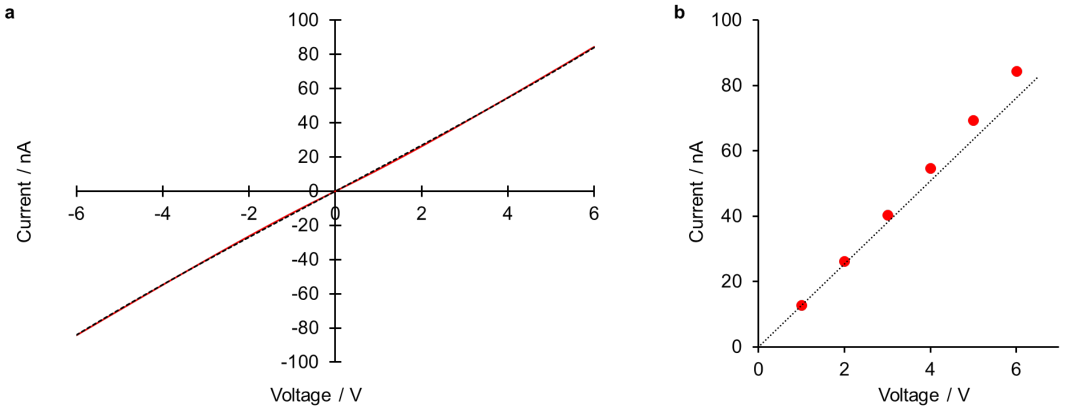

Supplementary Figure 15. Electrochemical conductivity of **2**. (**a**) Experimental *I-V* curve of 2 between −6 V and + 6 V (solid red line) and simulated *I-V* curve based on the hopping mechanism (black dotted line). (**b**) Comparison of *I-V* plot of **2** in **a** (red circle) and simulated linear *I-V* plot (black dotted line).

Supplementary Note 1.

The non-linear *I-V* curve was well-reproduced with the simulation based on the hopping conduction mechanism,[S1] in which the current *I* was given by the following equation;

$$I = I^{\circ}\ \{\exp(-nF\rho\phi/2RT) - \exp(nF\rho\phi\ /2RT)\}$$

where I°, ρ, and ϕ represent the intersite exchange current density, the fitting parameter, and average intersite voltage difference, respectively. In addition, I° was given by the following equation;

$$I^{\circ} = (10^3/6)\ nFC_{ox}C_{os}\delta\, k_{ex}$$

where C_{ox}, C_{red}, δ, and k_{ex} denote the concentration of the redox species, the average intersite distance, and the intersite electron transfer rate constant. Using the structural parameters of bis(terpyridine)metal(II) polymer in previous study[S2], the electron transfer rate constant was obtained as $k_{ex} = 4.4 \times 10^{-4}$ M^{-1} s^{-1}.

Q. Potential-dependent conductivity of **2**

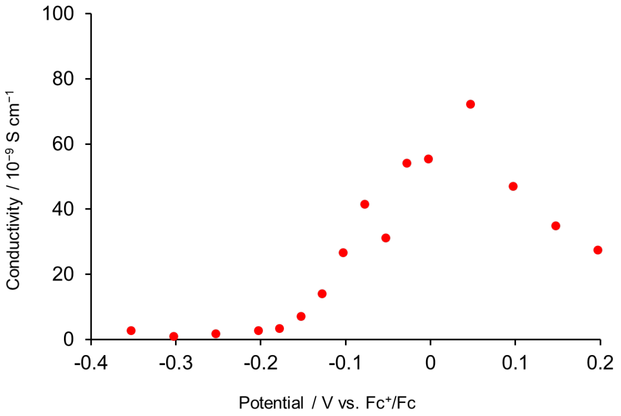

Supplementary Figure 16. Potential-dependent conductivity of **2**. (0.1 M *n*Bu_4NPF_6 in CH_3CN) Conductivity measurements were performed from the cathodic potential regions.

R. Electrochemistry of **2** and **3**

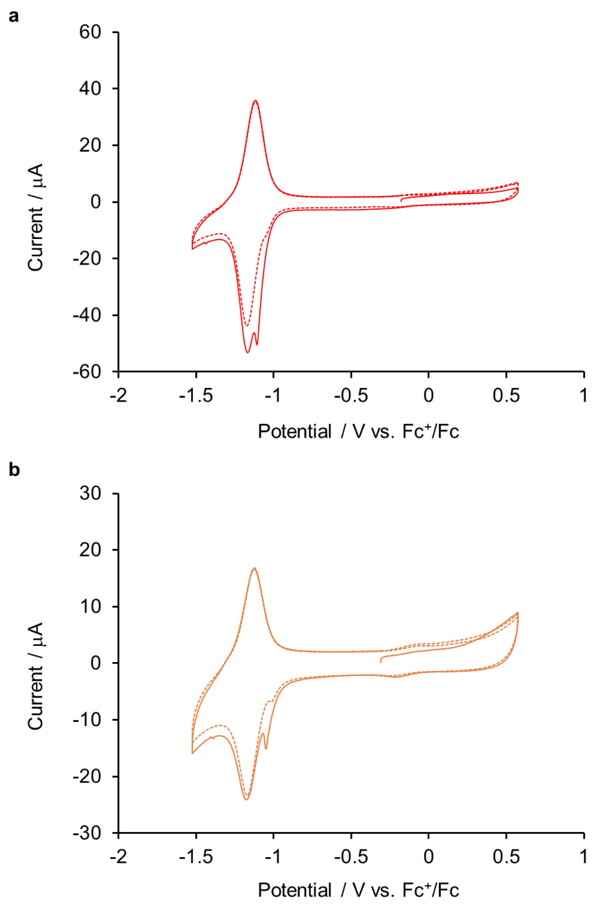

Supplementary Figure 17. Electrochemistry of **2** and **3**. Cyclic voltammograms of **2** (**a**) and **3** (**b**). (1 M *n*Bu_4NPF_6 in CH_3CN; Scan rate: 50 mV s^{-1}) The solid and dotted lines are the first and the second redox cycles, respectively.

S. Supplementary references

S1) Surridge, N. A. et al. Effects of Mixed-Valent Composition and Bathing Environment on Solid-State Electron Self-Exchanges in Osmium Bipyridine Redox Polymer Films. *J. Phys. Chem.* **96**, 962−970 (1992).

S2) Takada, K. Maeda, H. Nishihara, H. A Bis(terpyridine)nickel(II)-Based Coordination Nanosheet: A Redox-Active Material with Flexibility and Transparency. *J Inorg Organomet Polym* (2023). https://doi.org/10.1007/s10904-023-02921-4.
