# Supplementary material for: Modulation between capacitor and conductor for a redox-active 2D bis(terpyridine)cobalt(II) nanosheet via anion-exchange
Source: Commun Chem. 2024 Aug 22;7:186. doi: 10.1038/s42004-024-01274-4 (PMC11341730; doi:10.1038/s42004-024-01274-4)
Supplement: Supplementary file 3 — Description of Additional Supplementary Files [file 42004_2024_1274_MOESM3_ESM.pdf]

Description of Additional Supplementary Files

File name- Supplementary Data 1

File description- Dataset for FIgure 1-5 in the manuscript

File name- Supplementary Data 2

File description- Dataset for Supplementary Materials
